# Electrophoretic Deposition of Biocompatible and Bioactive Hydroxyapatite-Based Coatings on Titanium

**DOI:** 10.3390/ma14185391

**Published:** 2021-09-18

**Authors:** Marija Djošić, Ana Janković, Vesna Mišković-Stanković

**Affiliations:** 1 Institute for Technology of Nuclear and Other Mineral Raw Materials, Bulevar Franš d’Eperea 86, 11000 Belgrade, Serbia; mdjosic@yahoo.com; 2 Faculty of Technology and Metallurgy, University of Belgrade, Karnegijeva 4, 11000 Belgrade, Serbia; ajankovic@tmf.bg.ac.rs

**Keywords:** antibacterial coating, hydroxyapatite, silver, gentamicin, electrophoretic deposition, implants

## Abstract

Current trends in biomaterials science address the issue of integrating artificial materials as orthopedic or dental implants with biological materials, e.g., patients’ bone tissue. Problems arise due to the simple fact that any surface that promotes biointegration and facilitates osteointegration may also provide a good platform for the rapid growth of bacterial colonies. Infected implant surfaces easily lead to biofilm formation that poses a major healthcare concern since it could have destructive effects and ultimately endanger the patients’ life. As of late, research has centered on designing coatings that would eliminate possible infection but neglected to aid bone mineralization. Other strategies yielded surfaces that could promote osseointegration but failed to prevent microbial susceptibility. Needless to say, in order to assure prolonged implant functionality, both coating functions are indispensable and should be addressed simultaneously. This review summarizes progress in designing multifunctional implant coatings that serve as carriers of antibacterial agents with the primary intention of inhibiting bacterial growth on the implant-tissue interface, while still promoting osseointegration.

## 1. Introduction

Multifuncionality of medical devices is the key feature that enables their successful and prolonged usage. The primary role that osteoarticular implants need to meet is the complete recovery of lost function and successful implant fixation. However, there is mounting evidence that most of the problems associated with implant failure are infection at the implantation site and aseptic loosening [1]. Infection of the implantation site represents a major concern. After implantation procedures, patients are vulnerable and prone to infection due to the immune system efficacy being compromised by the presence of an implant and of the possibility that even a few bacteria could easily latch to the solid substrates, and unfortunately propagate easily to form a highly resistant biofilm. Once formed biofilms tend to be highly resistant to antibiotics, and could even cause infection in other tissues. Needless to say, the patients’ health could be severely threatened by these events that could have a fatal outcome. The essential problem of these medical procedures is a major threat that an occurrence of multidrug-resistant bacterial strains might bring. The havoc that methicillin-resistant *Staphylococcus aureus* can cause to the human body, is sadly evidenced in many medical reports worldwide. The statistical data citing infections of orthopedic implants depend on the type of implant device and procedure in question. The most widely cited infection rates are approx. 5% for orthopedic implants, but slightly higher (14%) for dental implants, including peri-implantitis or any dental implant infection. Osseointegration implies a firm, direct and lasting bonding of the periimplant bone to the implant. Incomplete osteointegration [2] represents a major culprit for aseptic loosening. The term osteointegration was originally used to describe the accommodation of titanium (Ti) dental implants [3] and the loosening of screw-shaped implant fixtures since there is no interposed tissue between fixture and the bone, but it could easily be applied to orthopedic implants. Osteointegration depends on how efficiently mechanical intertwining of the bone to the implant progresses after surgical positioning, and the cellular response at the bone-implant interface, processes that are crucial to ensure successful bone healing.

Exhaustive research exploring surface modification methods was pursued to either improve implant osteointegration or reduce bacterial infection. However, both of these issues need to be addressed at the same time. An example how these two approaches need to work synergistically instead of even being detrimental to the other is creating a rough implant surfaces that promote osteointegration. Unfortunately such materials are also ideal for increased bacterial attachment. Incorporating antibacterial agents might induce toxicity or impair normal metabolic functions.

Therefore, this review provides wider approach discussing in detail recent methods employed for implant surface modification enhancing osteoinductive and antibacterial properties at the same time.

## 2. Engineering Implant Surfaces to Prevent Microbial Adhesion and Infection

Despite all recent advancements in medicine, metallic implants are still the materials of choice in reconstruction surgery. Currently, titanium and titanium alloy (Ti6Al4V) [4,5], 316L stainless steel [6] and cobalt chromium alloys [7] are most commonly applied as metallic materials for biomedical applications. The desired properties of biomaterials encompass a wide range of material characteristics, e.g., good mechanical properties, high biocompatibility, high wear resistance, good corrosion resistance, non-toxicity (genotoxic nor cytotoxic) and osseointegration ability [8,9]. It is known that strong bonding between the implant and the surrounding tissue is required, reducing the risk of implant loosening. On the other side, release of toxic ions, degradation by corrosion or loosening of tissue bonding ability can be detected in metallic implants after prolonged use [10,11,12]. 

In tissue engineering, it is mandatory to obtain a bioactive material with defined mechanical and biological characteristics that mimic the natural tissue structure. The main inorganic part of natural bone, hydroxyapatite (HAP) (~65–70%) [13], is biocompatible, bioactive and osteoinductive, having a large surface area [14]. Having similar chemical composition to bones’ mineral phase, synthetic hydroxyapatite is commonly applied as a metallic implant surfaces’ modifier or as a bone filler material [15,16].

One solution to elevate osseointegration would be to assemble porous coatings on orthopedic implants, designed to facilitate osteoconduction. It is known that porous structures, in the form of individual, open or interconnected pores, have an extremely favorable effect on tissue integration with implants. Hydroxyapatite, having osteoconductive properties, is the material of choice in bone tissue engineering. One of the main goals in bone tissue engineering development is to combine high antibacterial activity while maintaining strong osteoconductivity. It was shown that hydroxyapatite coatings doped with Ag and Sr are able to increase defense against infections, promoting at the same time osteoblast proliferation and especially causing increased levels of alkaline phosphatase (ALP) biomarker of osteogenesis [17]. The coatings’ high antibacterial efficacy was observed after release of silver ions [18]. Different approach utilized osteoconductive coating materials that incorporated physically adsorbed bactericidal agents e.g., antimicrobial peptides (AMPs) [19]. These coatings provided platform for osteoblast attachment and at the same time showed osseointegration improvement in the conducted in vivo study of rabbit tibial model, compared to an uncoated Ti control. The AMP-based composite coating provided protection against *Staphylococcus aureus* and *Pseudomonas aeruginosa*. When the antibiotic norvancomycin was incorporated into the HAP coating, it provided antibacterial effects against the bacterial strain *S. aureus* with a documented short-term burst release, but without compromising osteogenic ability [20].

Natural or synthetic polymers are often used in bone tissue engineering [21,22]. The advantage of polymer application in reconstruction surgery, in the native or composite form, can be recognized in the possibility of producing structures with enhanced physical and mechanical properties, e.g., controlled degradation rates, porosity, enhanced biocompatibility etc. The wide field of biodegradable polymers’ applications includes dentistry [23,24], tissue engineering [25,26], drug delivery [27,28], orthopedic devices [29,30], artificial skin [31,32], and cardiovascular surgery [33,34]. The most used synthetic polymers in tissue reconstruction surgery are listed below:

- Poly(lactide-co-glycolide) (PLGA)—PLGA in combination with the natural polymer chitosan, applied as a stent coating, can reduce platelet adhesion [35], while the combination of PLGA with HAP and the antibiotic atorvastatin can be applicable in bone tissue engineering as injectable PLGA micro-particulate system [36].
- Poly(glycolic acid) (PGA)—in combination with hydroxyapatite, osteoblast differentiation and mineralized bone matrix formation can be increased after implantation of PGA/HAP composites [37].
- Polyether ether ketone (PEEK)—regardless of the fact that PEEK is a bioinert material, its excellent mechanical and chemical properties in addition to the fact that it does not induce positive/negative body reaction, make it a widely used material as a bone substitute, e.g., in dental implants, etc. Three-component coatings (PEEK, hydroxyapatite and chitosan) have efficient antibacterial properties against the microorganisms *E. coli* as well as *S. aureus* [38].
- Poly(lactic acid) (PLA)—biocompatible polymer often used because of its good biodegradability, high elastic modulus as well as the fact that the final degradation products of PLA are water and carbon dioxide, non-toxic or carcinogenic. Additionally, PLA has higher elastic modulus than natural cancellous bone [39]. Mechanical characteristics of composite coatings, e.g., elongation at break, substantially increased in hydroxyapatite nanorod network in the poly(lactic acid) [40].
- Poly-L-lactic acid (PLLA)—homogenous dispersion of hydroxyapatite nanoparticles and microcrystalline cellulose in a PLLA matrix led to the formation of nanocomposite material that is, according to the composition, structure and mechanical characteristics, comparable with the trabecular bone [41].
- Poly-caprolactone (PCL)—can be used as a drug delivery device and biomaterial for regenerative medicine. PCL can be employed as the binding agent for PCL-hydroxyapatite scaffold preparation. Poly-caprolactone/hydroxyapatite scaffold with heparin sulfate showed positive effects on the differentiation of osteoblasts, accelerating the repair of biological bone defects [42].
- Poly(vinyl alcohol) (PVA)—is known as a flexible, biocompatible, and biodegradable polymer, having a high tensile strength. PVA has the ability to form composites with chitosan, leading to formation of nanofibrous polymer matrix for hydroxyapatite incorporation, mimicking native extracellular matrix for bone tissue repairing. It was demonstrated that the nanofibrous scaffold of PVA/chitosan/hydroxyapatite provides versatile surface for attachment and proliferation of the osteoblast cells [43].
- Polymethyl methacrylate (PMMA) is an acrylic material that is widely used in dentistry due to its high tensile and flexural modulus. Addition of hydroxyapatite into PMMA leads to formation of composite material with improved characteristics, e.g., the density, and tensile and flexural modulus of PMMA increased significantly after incorporation of hydroxyapatite [44].
- Natural polymers, such as collagen, fibrinogen, hyaluronic acid, elastin, alginate, chondroitin sulfate, lignin, and chitosan can be used to obtain materials for hard and soft tissue repair [45]. The efficiency of the natural polymers’ application in tissue engineering is reflected in the possibilities to be recognized by host cells, due to the specific amino-acid sequences in their structure [46]. Additionally, the polymers are able to promote tissue healing and integration, through the induction of biochemical signals that trigger cell migration, proliferation, and differentiation [47]. Collagen sponge material, for example, can provide cell growth and promote the nutrients absorption. In clinical practice, the application of the collagen sponge culture system combined with the Rotary Cell Culture System is a promising tool for tissue engineering [48]. Fibrinogen is the major plasma glycoprotein coagulation factor. Its role is to facilitate the adhesion, spreading and aggregation of the cells. Addition of fibrinogen to the hydroxyapatite nanoparticles improves the cell adhesion [49]. The two predominant proteins in the body, responsible for modulating biological and mechanical properties of tissues, are elastin and collagen [50]. Elastin, found in connective tissue, is a bioactive protein, very often used in tissue engineering. A composite of elastin and collagen, can be applicable in vascular tissue engineering, e.g., tubular polymer scaffolds. Since the blood vessels are predominantly fibrous, composed of collagen and elastin, the combination of these two polymers with polyurethane, was employed in the production of fibrous scaffolds. The obtained scaffold was hydrophilic and cell viability was proven for 72 h [51]. The natural ionic mucopolysaccharide with linear structure hyaluronic acid, possesses multiple active groups (carboxyl, hydroxyl and amino groups) that can be further chemically modified, expanding the applications of hyaluronic acid in the biomedical field [52]. Oxidized hyaluronic acid, due to its lower cytotoxicity, was investigated as a potential substitute of glutaraldehyde, as a fixation component in the abdominal wall repair surgery [53]. Low toxicity, degradability in the physiologic conditions, high affinity to calcium ions, and hydrophilic nature, make alginate as potent candidate material in tissue engineering [54]. Microencapsulation of hydroxyapatite in scaffolds obtained by the combination of alginate and gelatin, results in a promising material to induce osteogenic differentiation and to improve cell proliferation [55]. Chondroitin sulfate is known to facilitate cell proliferation and for having antithrombogenic activity. By combination of chondroitin sulfate and collagen, vascular scaffold with antithrombosis and endothelialization function can be obtained [56]. One of the most widely used natural polymers, chitosan (CS), possesses all the main characteristics required for biomedical application as a drug carrier, or a component of the biocompatible coating or repair of hard and soft tissues [57]. The most valuable characteristic of CS, as a biomaterial approved by Food and Drug Administration [58,59] are: biocompatibility [60], biodegradability [61], antibacterial activity [62,63], and high potential in drug delivery systems [64,65]. The tissue response upon implantation is reduced to a minimum due to CS presence, thanks to its hydrophilic surface that improves cell adhesion, differentiation and proliferation [66]. Additionally, it was shown that, during in vivo investigation, the degradation of CS occurs under the influence of some proteases (mostly lysozymes), leading to formation of non-harmful oligosaccharides [67]. As a drug carrier in orthopedic implants, chitosan can be employed in local drug administration of antibiotics. In that way, it is possible to preserve the therapeutic effect of antibiotic with lower administered dose. It was shown that gentamicin release from the composite hydroxyapatite/chitosan/gentamicin coating included the initial burst-release effect, meaning that more than 50% of the total amount of pre-loaded antibiotic was released during the first 7 days. An additional advantage of chitosan presence in composite coating can be observed through the slower release of gentamicin during the next 14 days. Prolonged gentamicin release from the hydroxyapatite/chitosan/gentamicin composite coatings makes these coatings great candidates for potential application in the treatment of orthopedic infections [68]. Composite coatings based on chitosan and hydroxyapatite show excellent biocompatibility, which was confirmed through the formation of new hydroxyapatite layer after only 7 days of immersion in simulated body fluid (SBF) [68,69]. Above everything mentioned before, chitosan has very high position in the field of biomaterials production due to its exceptional properties of easy film forming ability [57,70]. Chitosan, as a cationic polysaccharide, can be used in the electrophoretic deposition process (EPD), one of the most attractive techniques for bioactive coatings production [68,69,71,72,73,74,75,76,77].
- Lignin (Lig) is a polyphenolic polymer, originated from the various natural sources [78,79,80]. According to the different processing methods used during production, several different types of lignin can be distinguished, e.g., kraft lignin, organosolv lignin, soda lignins [81]. The existence of different lignin types has limited its application in the biomedical fields. Despite these drawbacks, lignin possesses some unique properties, making it suitable for biomedical application, e.g., antioxidant, antibacterial, thermal stability, anti-ultraviolet protection, antigenotoxic, anticarcinogenic, antimutagenic and biocompatibility [80,82]. Organosolv lignin represents the purest type of lignin, extracted from natural sources (hardwood, softwood, plant crop) by solvent precipitation [73]. Lignin found the application as a drug nanocarrier [83,84], as well as in preparation of bioactive composite coatings [73,74,75] and tissue scaffolds [85]. Composite coatings based on lignin and hydroxyapatite with the addition of antimicrobial agent, such as silver, show excellent biocompatibility after SBF immersion. Namely, layer of newly grown hydroxyapatite can be obtained after 7 days of immersion [73].

Various kinds of antibiotics are well known in the medical treatment of a wide range of bacterial infections. In some cases, e.g., orthopedic surgery, it is absolutely necessary to ensure a high local concentration of the drug, with the aim to prevent initial biofilm formation, as explained in detail above. Therefore, considerable attention was focused on antibiotic-loaded composites obtained by EPD technique. Thorough study on EPD deposited graphene oxide/chitosan (GO/CS) films with effectively incorporated vancomycin antibiotic it has been shown that novel drug-eluting composite coatings were formed [86] that exhibited elevated amounts of incorporated antibiotic as a consequence of the GO nanosheets’ influence on the polymer matrix. The encapsulated drug was released swiftly, followed by a slower rate for up to 4 weeks. The antibacterial effect was tested against *S. aureus* and it was estimated that for drug concentrations >0.5 g/L, no bacteria remained. A biocompatibility assay utilizing a human osteosarcoma cell line showed that the cytotoxicity of composites varied based on the concentration of the GO component. Interestingly, levofloxacin was successfully incorporated by EPD using a layer-by-layer approach [87]. In a similar study of titanium implants coated by poly(di(ethylene glycol) methyl ether methacrylate) brushes, with temperature-triggered release of levofloxacin, antibiotic levels reached minimal inhibitory concentration (MIC) values for *S. aureus* American Type Culture Collection (ATCC) 13709 near the implant surface [88]. EPD was also used for depositing hydroxyapatite nanoparticles loaded with gentamicin sulfate and ciprofloxacin [89] that exhibited bioactivity by enhancing the precipitation of ions from the SBF solution, thus mimicking in vitro implant biomineralization. At the same time, the coatings were very efficient against *P. aeruginosa* bacteria. In a comparative study of carbonated hydroxyapatite coatings on titanium implants loaded with antibiotics—amoxicillin, cephalothin, vancomycin, carbenicillin, tobramycin, gentamicin and cefamandol—different release patterns and antibacterial activity were observed depending on their chemical structure [90]. Tobramycin and gentamicin, lacking carboxylic acid groups, were quickly released from the coating, whereas cephalothin was efficiently incorporated, and therefore it had sustained release and was highly active against the tested organism *S. aureus*. When ampicillin was incorporated into a chitosan-based composite coating prepared via EPD on a Ti substrate, it showed highly sustained 28-day release behavior with no initial burst effects and promoted adhesion and proliferation of pre-osteoblast cells (MC3T3-E1) cultured on the coating’s surface [91]. Gentamicin incorporated into HAP/CS coating via a single-step EPD route yielded highly antibacterial coatings on Ti that showed elevated activity against the bacterial strains *S. aureus* and *E. coli* [72]. In vitro cytotoxicity assessment by tetrazolium salt MTT (3-(4,5-dimethylthiazol-2-yl)-2,5-diphenyl tetrazolium bromide) testing proved the coatings were not cytotoxic when tested in the L929 (mice fibroblasts) and MRC-5 (human fibroblasts) cell lines.

Having in mind the plethora of publications in this field, we narrow down our efforts to the investigation of multifunctional chemical coatings with two representative antibacterial agents, silver embedded in the hydroxyapatite (Ag/HAP) and hydroxyapatite/lignin (Ag/HAP/Lig) coatings and antibiotic gentamicin, non-specifically bound to composite hydroxyapatite/chitosan (HAP/CS/Gent) and hydroxyapatite/chitosan/graphene (HAP/CS/Gr/Gent), all deposited on Ti using EPD. During last decade, interest in EPD application for the production of coatings has increased [92,93,94,95,96,97,98,99]. The main advantage of EPD, compared to the other particle-processing techniques for materials production, is its single-step deposition of composite coatings from multi-component deposition bath [69,73,100,101] at room temperature. Moreover, application of EPD allows the production of uniform coatings with desired morphology and controlled coating thickness on substrates of different shapes and inner surfaces by varying deposition parameters.

## 3. Engineering Bactericidal Surfaces by Incorporating Silver

Bactericidal coatings are a potent tool used to halt the infection directly, and may eliminate such threats altogether, acting in sync with a healthy immune system. Silver is the most common bactericidal agent currently used in research studies and in clinical practice. However, exceeding the optimal silver concentration could be deleterious to bone growth, emphasizing the delicate balance between antimicrobial functionality and osseointegration and therefore finely tuned synthesis method such as EPD could help in mastering the right composition.

### 3.1. Hydroxyapatite Coatings with Silver and Lignin

Silver resurfaced as a therapeutic option due to alarming warnings of bacterial resistance to commonly used antibiotics. This renewed interest prompted researchers to explore the antimicrobial properties of silver and broadened its potential applications. The simplicity of the system that relies on silver-doped hydroxyapatite powder provided valuable data for the release of inorganic antimicrobial agent. As doping agents, silver and silver ions inhibit bacterial attachment onto biomaterial surfaces almost immediately upon contact and act against a broad spectrum of bacteria. Silver can be used for doping different materials (ceramics, metals, polymers) and particularly interesting—doping of hydroxyapatite. Lignin, known for its antioxidant and antimicrobial properties, was added to Ag/HAP composite, trying to improve the coatings structure to prompt osteogenesis.

Complex molecule like lignin carries many functional groups, but phenolic hydroxyl groups represent the most reactive chemical sites [102,103]. Depending on the plant origin and the pulping processes, lignins show varied ratios of methoxyl, carbonyl and carboxyl groups present. According to the proposed mechanism [75] the electrophoretic deposition of Ag/HAP and Ag/HAP/Lig particles occurs in several stages. The first stage occurs when the charged particles attract oppositely charged ions (i.e., counterions). The positively charged Ag/HAP and Ag/HAP/Lig particles migrate toward the cathode where they discharge. In general, when the particle is sufficiently close to the cathode surface, attractive forces are prevalent and cause coagulation/deposition. Simultaneously, hydrogen evolves on the cathode (Equation (1)) and oxygen on the anode (Equation (2)):

cathode: 2H_2_O + 2e^−^ → H_2_ + 2OH^−^ (1)

anode: 2H_2_O → 4H^+^ + O_2_ + 4e^−^ (2)

The evolved hydrogen leaves the cathode through the deposited Ag/HAP and Ag/HAP/Lig thus forming coatings porous structure.

EPD was performed from absolute ethanol suspensions composed of 1 wt% of nanosized Ag/HAP powder (for Ag/HAP coatings) or 1 wt% of nanosized Ag/HAP powder and 0.01 wt% of lignin (for Ag/HAP/Lig coatings). The three-electrode cell arrangement was utilized with titanium plate as a working electrode and two platinum panels as counter electrodes. The deposition was performed at room temperature applying voltage of 60 V and 45 s deposition time [73,74,76,104].

**Figure 1:**
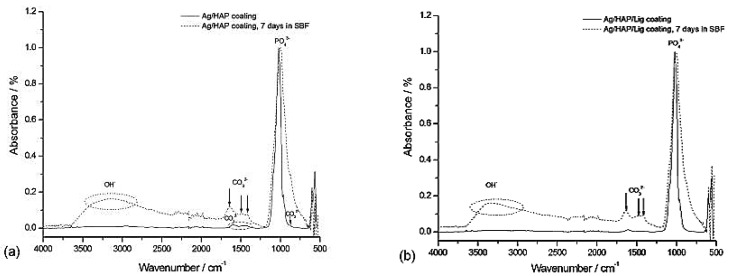
FT-IR spectra of Ag/HAP (**a**) and Ag/HAP/Lig (**b**) coatings on titanium before and after 7 days of immersion in SBF at 37 °C. Reprinted with permission from [74]. Copyright (2013) American Chemical Society.

The bioactivity of Ag/HAP and Ag/HAP/Lig coatings, before and after immersion in SBF, was investigated by FT-IR, XRD, SEM and electrochemical impedance spectroscopy (EIS) analyses [73,74,76,104]. In FT-IR spectral bands for as-deposited Ag/HAP and Ag/HAP/Lig coatings, i.e., before immersion in SBF (Figure 1a,b, respectively), the most intensive vibrational bands, assigned to the phosphate (PO_4_^3−^) group, were observed at 960, 1016 and 1089 cm^−1^, confirming the presence of HAP in both coatings. Bands at 627 and 3573 cm^−1^ indicate structural OH^-^ groups, additionally proving the presence of HAP [105]. Low-intensity bands appeared in the region from 1400–1585 cm^−1^ as well as band at 875 cm^−1^ and can be attributed to carbonate ions. In FT-IR spectra for both coatings, after immersion in SBF for seven days, the broad band that appeared at 3382 cm^−1^ originated from the –OH stretching mode, indicating the bone like apatite formation [106]. The triplet of bands at 1640, 1476 and 1420 cm^−1^ could be assigned to the carbonate groups, pointing to the substitution of the PO_4_^3−^ (from the HAP lattice) by CO_3_^2−^ groups. The position of triplet bands revealed the presence of B-type carbonated apatite [105]. Higher concentration of hydroxyl and phosphate groups, observed from more intensive bands for both coatings after immersion in SBF, provide negative surface potential required for hydroxyapatite nucleation, indicating the bioactivity of both coatings. According to the literature, hydroxyapatite in bone mineral belongs to the carbonate-substituted apatites, i.e., it differs from stoichiometric HAP, and is well known for its bioactivity [107].

**Figure 2:**
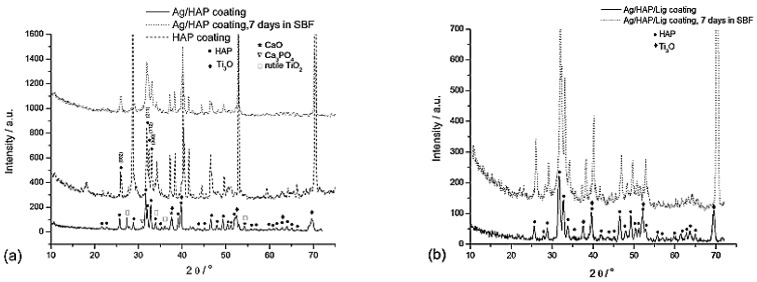
XRD patterns of Ag/HAP (**a**) and Ag/HAP/Lig (**b**) coatings on titanium before and after 7 days of immersion in SBF at 37 °C. Adapted from with permission from MDPI [73]. Adapted with permission from [74]. Copyright (2013) American Chemical Society.

XRD patterns for Ag/HAP (Figure 2a) and Ag/HAP/Lig (Figure 2b) coatings after immersion in SBF for 7 days, revealed the shifting of hydroxyapatite characteristic diffraction maxima toward higher angles as a consequence of CO_3_^2−^ ions incorporation in the HAP lattice, i.e., the growth of new carbonate HAP onto the coating surface. Crystallite domain size changed for both coatings after immersion in SBF, i.e., from 35.2 nm to 20.2 nm for Ag/HAP coating and from 20.8 nm to 22.0 nm for Ag/HAP/Lig coating [73,74], before and after immersion, respectively, proving the bone-like apatite formation through the incorporation of carbonate ions into the apatite lattice by replacement of hydroxyl or phosphate groups [108,109]. The growth of new carbonate-substituted hydroxyapatite layer is highly beneficial due to its similarity to the actual bone tissue [110].

**Figure 3:**
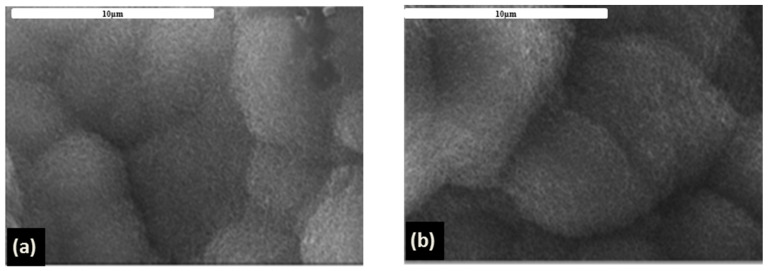
FE-SEM microphotographs of Ag/HAP (**a**) and Ag/HAP/Lig coating (**b**) after immersion in SBF at 37 °C.

After just 7 days of immersion in SBF, on the top of both Ag/HAP and Ag/HAP/Lig coatings (Figure 3a,b, respectively) new carbonate-substituted HAP was observed. The mechanism of biomimetic apatite formation in SBF occurs due to the high concentration of OH^−^ and PO_4_^3−^ groups on the HAP surface i.e., a negatively charged surface, specifically through interaction with the Ca^2+^ ions from the surrounding fluid, leading to the formation of amorphous Ca-rich apatite. Negative PO_4_^3−^ ions, from the SBF, interacted with positively charged surface, forming Ca-poor apatite. Once formed through the gradual crystallization of Ca-poor apatite, bonelike apatite grows spontaneously, consuming the Ca^2+^ and PO_4_^3−^ ions. Having in mind that the composition of the SBF solution includes a significant number of different inorganic salts, incorporation of minor ions (Na^+^, Mg^2+^, CO_3_^2−^) in apatite structure occurs, leads to the formation of biomimetic apatite quite similar to the apatite in natural bone minerals [111].

Increase in pore resistance, *R*_p,_ determined from EIS analysis [74] for Ag/HAP and Ag/HAP/Lig coatings (Table 1), indicated the new apatite growth after prolonged time in SBF, confirming previously represented results, obtained by XRD (Philips PW 1051 Powder Diffractometer, Amsterdam, The Netherlands), FTIR (Spectrum™ 400 Perkin Elmer Infrared Spectrometer, Waltham, MA, USA) and FE-SEM (JEOL JSM-5800, Akishima, Tokyo, Japan).

Bearing in mind that new carbonate substituted HAP was formed on the top of both Ag/HAP and Ag/HAP/Lig coatings, it could be proposed that coatings’ surfaces enabled the nucleation and growth of a new biomimetic HAP layer, thus inducing stable bonding to the bone in the human body, desired characteristics for potential application as a biomaterial [112].

#### Silver Release

The antibacterial efficiency of a coated material is measured by the release of antimicrobial agent into the surrounding physiological environment [113]. The coatings incorporating silver ions are designed in such a way as to provide high initial concentration of antimicrobial. The idea is to prevent the initial adherence of bacteria, crucial during the early post-implantation period [114]. After this critical period, continuous silver ion release is preferred in order to definitely avoid biofilm formation. Released silver ion concentration from the Ag/HAP/Lig coating during 10 days at 37 °C in SBF is shown in Table 2. The cumulative silver ion concentration released during this time period was measured to be 1.704 ppm [76]. Previously reported literature data stated that minimum effective concentration is 0.1 ppb and the maximum cytotoxic concentration toward human cells is 10 ppm [114], therefore the reported values were within these limits. The silver release from Ag/HAP/Lig coating was within the range of initial antibacterial concentration, which was found to be 56 ppb.

The presence of potent antibacterial agents such as silver and silver ions in the composites provided the desired antibacterial effect, therefore both Ag/HAP and Ag/HAP/Lig coatings had a swift antibacterial effect against *S. aureus* TL (Table 3). After just 1 h of incubation cell growth declined by two logarithmic units compared to the initial cell counts in suspensions (percentage reduction was 98.17% and 97.67%, respectively) [76]. Based on the data, the eluted silver ion concentration was 0.455 ppm after 1 h (Table 2), well balanced amount to reach antibacterial effect without evoking cytotoxicity. The antimicrobial efficiency of both coatings exhibited high reduction of *S. aureus* TL strain, as after 24 h, no visible colonies were detected in the aliquots taken from the suspensions, thus satisfactory protection against infection would be assumed. Released quantity of silver ion assured imminent drop in CFU counts after 1 h inoculation. Such a strong bactericidal effect would provide protection against biofilm formation.

Cytotoxicity was determined by reduction of a yellow tetrazolium salt (3-(4,5-dimethylthiazol-2-yl)-2,5-diphenyltetrazolium bromide (MTT)) to purple formazan crystals by metabolically active cells, in this case by peripheral blood mononuclear cells (PBMC) cells and PHA-stimulated PBMC cells (Table 3) [76]. PBMC are the principal populations of the human immune system cells, predominantly lymphocytes and monocytes [115]. The absolute requirement for any type of biomaterial as part of a medical device would be lack of any toxic effects to the surrounding tissue as well as against healthy immunocompetent PBMCs.

Ag/HAP and Ag/HAP/Lig coatings mildly affected survival rates of healthy immunocompetent PBMC, unstimulated and stimulated to proliferation, compared to the control cell sample (100% survival). According to the scale of cytotoxicity for materials (S, cell viability >90%—noncytotoxic, 60–90%—slightly cytotoxic, 30–60%—moderately cytotoxic, ≤30%—cytotoxic), adapted from Sjogren et al. [116], and adopted classification, Ag/HAP and Ag/HAP/Lig coatings were classified as non-cytotoxic against targeted PBMC.

## 4. Designing Coatings with Long-Term Release of Antibiotics

Very often, in modern medicine, local administration of antibiotics or supplements is used, as a promising alternative to the traditional way of systemic application of antibiotic therapy. Drug carriers could be attached directly to the implant providing local antibiotic release. On the other hand, antibiotics can be incorporated into the surface of the implant itself. Such a strategy depends on the type of antibiotic and its ability to remain functional in such conditions.

### 4.1. Hydroxyapatite Coatings with Gentamicin, Chitosan and Graphene

The choice was made to employ a strategy that utilizes a carrier polymeric matrix e.g., chitosan to deliver in situ chosen antibiotic-gentamicin. Implementation of gentamicin sulfate, as an antimicrobial agent, attracted extreme interest in the biomaterials’ field, due to gentamicin broad antibacterial spectrum of action [117,118,119,120,121,122,123]. With the aim to obtain antibacterial and biocompatible coatings on titanium, containing gentamicin as antimicrobial agents, EPD was employed [124,125,126,127].

Mechanism of EPD formation of chitosan coatings is determined by its amino groups, i.e., chitosan shows pH-dependent solubility, and allows processing from the aqueous suspension [128]. At pH values below pKa (pKa of chitosan is ~6.3), primary amino groups of chitosan become protonated, making chitosan positively charged and water-soluble in acidic medium (Equation (3)) [129,130,131]:

CS-NH_2_ + H_3_O^+^ → CS-NH_3_^+^ + H_2_O (3)

Gentamicin (Gent), as an aminoglycoside antibiotic, has high water solubility and is completely stable in the wide pH range (e.g., 2.0–10.0). Additionally, interaction of CS and HAP occurs through the formation of hydrogen bonds, i.e., amino and hydroxyl groups in CS and Gent interact with hydroxyl groups in HAP. 

The mechanism of coatings electrophoretic deposition from the aqueous suspension under constant voltage, includes electrolysis of water hydrogen and oxygen evolution on the electrodes (Equations (1) and (2)), causing the increase in the local pH at the cathode and coating deposition on the cathode. Protonated CS molecules (Equation (3)) migrated toward the cathode, reacted with hydroxyl ions and attached to the cathode surface as insoluble coating (Equation (4)):

CS−NH_3_^+^ + OH^−^ → CS−NH_2_ + H_2_O (4)

In acidic solution, protonation of the HAP surface occurs. Driven by the electric field, positively charged HAP particles move to the cathode (Ti plate), are deprotonated and form coatings simultaneously with CS coagulation. In the case of antibiotic-loaded coating, HAP/CS/Gent, positively charged Gent migrated toward the cathode as well, where it deprotonated and formed a composite coating with HAP and CS on a Ti substrate.

Composite coatings on Ti were electrophoretically deposited from two different aqueous suspensions, HAP/CS/Gent (1 wt.% of HAP, 0.05 wt.% of CS and 0.1 wt.% of Gent) and HAP/CS/Gr/Gent (1 wt.% of HAP, 0.05 wt.% of CS, 0.01 wt.% of Gr, and 0.1 wt.% of Gent). A three-electrode cell arrangement, e.g., titanium plate as a working electrode and two Pt panels as counter electrodes were employed to deposit composite coatings at room temperature, constant voltage of 5 V and deposition time of 12 min [68,71,72].

**Figure 4:**
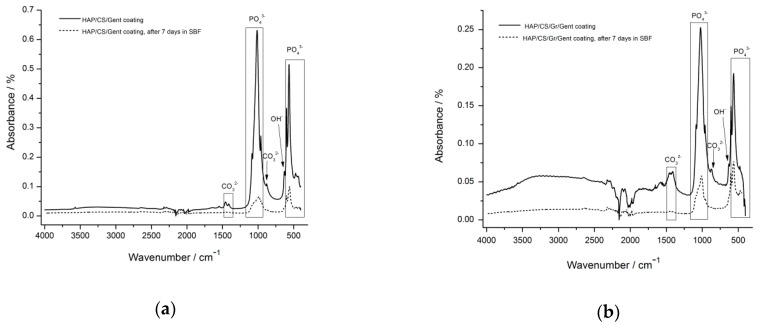
FT-IR spectra of HAP/CS/Gent (**a**) and HAP/CS/Gr/Gent (**b**) coatings before and after immersion in SBF at 37 °C.

With the aim to investigate the bioactivity of composite HAP/CS/Gent and HAP/CS/Gr/Gent coatings, FT-IR, XRD, FE-SEM and EIS analyses was performed for coatings before and after immersion in SBF. The most intensive bands in the FT-IR spectra, corresponding to phosphate, hydroxyl and carbonate groups, as well as the bands proving the presence of chitosan (Figure 4), can be detected in both HAP/CS/Gent and HAP/CS/Gr/Gent coatings before immersion in SBF [71,72]. Phosphate groups in the FT-IR spectra for HAP/CS/Gent coating before immersion in SBF (Figure 4a) are positioned at 472, 565, 600, 961, 1014 and 1086 cm^−1^, as well as for HAP/CS/Gr/Gent coating (Figure 4b) [71,72]. Bands at 629 and 634 cm^−1^, detected for both coatings, can be assigned to structural OH^−^ bending in hydroxyapatite [105], while OH^−^ stretching from hydroxyapatite structure can be detected at 3570 cm^−1^. Bands positioned at 879, 1412 and 1456 cm^−1^ for HAP/CS/Gent and 878, 1411 and 1458 cm^−1^ for HAP/CS/Gr/Gent coating (Figure 4a,b), indicate the presence of CO_3_^2−^ groups belonging to the carbonate-substituted HAP. According to the position of carbonate bands, HAP in both composite coatings belongs to so-called “AB-type” carbonate-substituted HAP, known for its excellent similarity to the natural bone and bioactivity [71,72,108,132]. Chitosan in the HAP/CS/Gent coating (Figure 4a) was confirmed by an amide I band at 1648 cm^−1^ (stretching of the C=O group) and amide II band at 1548 cm^−1^ (N–H bending). Almost the same position of FT-IR bands for CS can be observed in the spectra for HAP/CS/Gr/Gent coating (Figure 4b). New band at 1640 cm^−1^, was attributed to N–H bending vibration in primary aromatic amines, confirming Gent incorporation inside the composite coatings [69,71,72,133,134]. The interaction of CS and HAP was established by hydrogen bonding between hydroxyapatite hydroxyl groups and the hydroxyl and amino groups in CS. The graphene presence in HAP/CS/Gr/Gent was confirmed through the existence of a band at 1559 cm^−1^ (Gr skeletal vibration) [71].

FT-IR spectra obtained after 7-day immersion of HAP/CS/Gent and HAP/CS/ Gr/Gent coatings in simulated body fluid at 37 °C (Figure 4a,b, respectively), revealed the most intense bands at 960−1200 cm^−1^ and 500–600 cm^−1^ for phosphate groups, confirming the formation of new calcium phosphate phases [68,135]. Comparing the FT-IR before and after immersion in simulated body fluid for both HAP/CS/Gent and HAP/CS/Gr/Gent coatings, the absence of band at 3570 cm^−1^ (OH^−^ stretching from the HAP), can be observed [68,72], suggesting the formation of carbonate substituted HAP [110]. Additionally, the position of carbonate bands in FT-IR spectra at 878 cm^−1^ and in the region 1400–1550 cm^−1^ revealed the presence of “AB-type” substitution in HAP [72,136], similar to the biological apatites’ structure [108].

**Figure 5:**
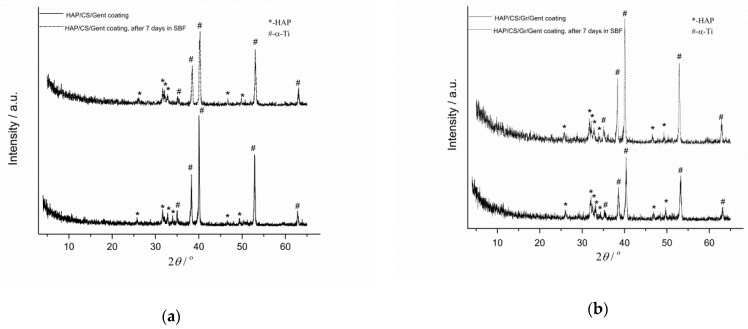
XRD pattern of HAP/CS/Gent (**a**) and HAP/CS/Gr/Gent (**b**) coatings before and after immersion in SBF at 37 °C Reprinted with permission from [72]. Copyright (2018) American Chemical Society. Reprinted with permission from [68]. Copyright (2020) American Chemical Society. Reprinted from [71]. Copyright (2020), with permission from Wiley.

Characteristic diffraction maxima for HAP and Ti (reflecting substrate) can be detected in XRD patterns for HAP/CS/Gent and HAP/CS/Gr/Gent coatings before and after immersion in SBF (Figure 5a,b, respectively) [68,71,72]. XRD patterns of newly formed layer obtained on the top of both coatings after immersion in buffered fluid (7 days at 37 °C) revealed the biomimetic HAP formation (crystallite domain size of HAP/CS/Gent coating changed from 39.7 nm to 37.4 nm, and from 31.1 nm to 36.0 nm, for the HAP/CS/Gr/Gent coating, before and after immersion, respectively). The Ca/P atomic ratios for HAP/CS/Gent and HAP/CS/Gr/Gent coatings, based on the XPS results 1.20 and 1.27, respectively [71,72] revealed the existence of calcium-deficient hydroxyapatite, i.e., carbonate-substituted HAP with the Ca/P ratio lower than 1.67 [137,138].

**Figure 6:**
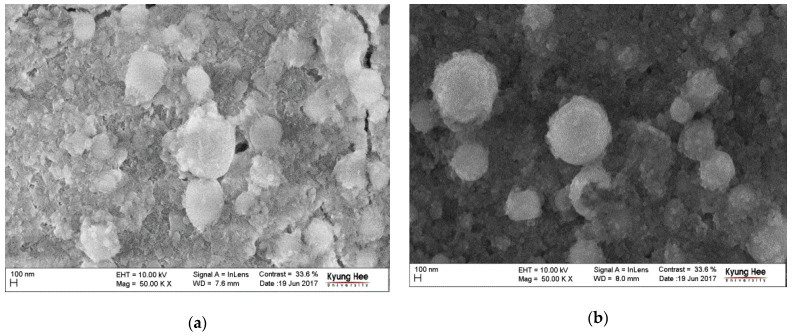
FE-SEM microphotographs of (**a**) HAP/CS/Gent and (**b**) HAP/CS/Gr/Gent coatings after immersion in SBF at 37 °C.

FE-SEM microphotographs of HAP/CS/Gent and HAP/CS/Gr/Gent coatings, after immersion in SBF (Figure 6a,b, respectively), present the newly formed biomimetic HAP with spherical agglomerates.

**Figure 7:**
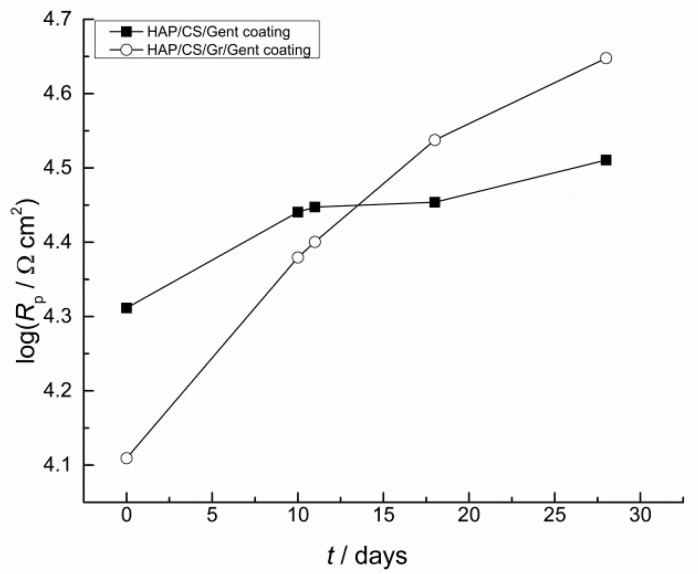
Time dependence of coating pore resistance, *R*_p_, for HAP/CS/Gent and HAP/CS/Gr/Gent coatings during exposure to SBF at 37 °C.

EIS was employed to confirm the HAP/CS/Gent and HAP/CS/Gr/Gent coatings’ bioactivity during extended submission in SBF [68]. The increase in coating pore resistance, *R*_p_ (Figure 7) confirmed the growth of newly formed HAP on the top of both coatings, as it was previously presented (Figure 4, Figure 5 and Figure 6).

#### Gentamicin Release

Local drug administration complements systemic drug therapies, mandatory with any implant-related procedures. Such delivery route supplies the opportunity to administer a lower dose of the drug without compromising the desired therapeutic effect. When devising a drug-eluting orthopedic device, many aspects must be considered, e.g., coating composition, porosity and thickness, as well as the nature of the release medium. Both the amount of loaded gentamicin in the composite coatings and the corresponding release profile were obtained by high-performance liquid chromatography coupled with mass spectrometry (HPLC-MS) [68] (Table 4).

The generated release profiles verified the initial 7-day burst effect, releasing more than 50% of gentamicin within this timeframe. This was followed by a slower release dynamic, with only 13% and 9% additional gentamicin release until 21st day from HAP/CS/Gent HAP/CS/Gr/Gent coatings, respectively. Judging by the obtained drug release capabilities, the obtained composite coatings could serve as long-term drug-delivery systems for treating and preventing bone infections.

**Figure 8:**
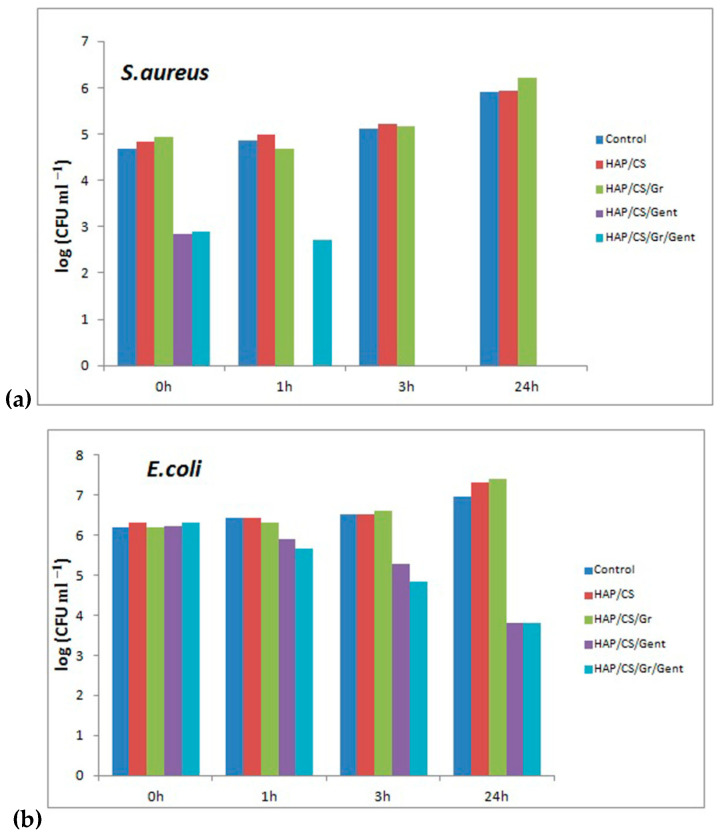
Reduction of viable cell number of: (**a**) *S. aureus* TL and (**b**) *E. coli* ATCC 25922 after contact with HAP/CS/, HAP/CS/Gr, HAP/CS/Gent, and HAP/CS/Gr/Gent coatings for 0, 1, 3 and 24 h in PB as compared to the control. Adapted with permission from [72]. Copyright (2018) American Chemical Society. Adapted from [71]. Copyright (2020), with permission from Wiley.

Antibacterial activity of HAP/CS/Gent and HAP/CS/Gr/Gent coatings, as well as HAP/CS and HAP/CS/Gr coatings without added antibiotic, against *S. aureus* TL and *E. coli* ATCC 25922 in modified phosphate buffered saline (PBS) is presented in Figure 8 [68,71,72]. Although the CS antimicrobial effect is well documented and the same applies for antibacterial features of graphene and all graphene-related species, expected synergistic effect in the composites could not be verified against these two chosen bacterial strains under these specific conditions.

Overall, very diverse effects of the coating composite materials were observed depending on the bacterial species. For the *S. aureus*, in the case of HAP/CS/Gent the drug release seems to be immediate and the bacterial counts drop to zero after a 1 h long incubation. HAP/CS/Gr/Gent coating also expressed strong bactericidal effects after short-term inoculation. After the initial contact, the adhered molecules of gentamicin are readily released and reduce the initial bacterial cells count by 2 logarithmic units. Even so, drug release seems delayed during the first hour, pointing to the strong non-specific interactions of gentamicin with both chitosan and graphene. For HAP/CS/Gr/Gent coating complete reduction of *S. aureus* bacterial cells occurred within 3 h of inoculation (Figure 8a). Therefore, both HAP/CS/Gent and HAP/CS/Gr/Gent composite coatings exert strong bactericidal against *S. aureus* TL.

Retained antibacterial effect of HAP/CS/Gent HAP/CS/Gr/Gent coatings was evident against *E. coli* (Figure 8b). Immediately upon inoculation, the drug release barely affected the bacteria i.e., they expressed low sensitivity towards the antibiotic. During total experimental period (24 h post incubation) surviving *E. coli* cell counts substantially declined. The activity of both HAP/CS/Gr and HAP/CS/Gr/Gent coatings could be classified as bacteriostatic, as they caused less than 3 logarithmic units reduction in bacterial cells.

**Figure 9:**
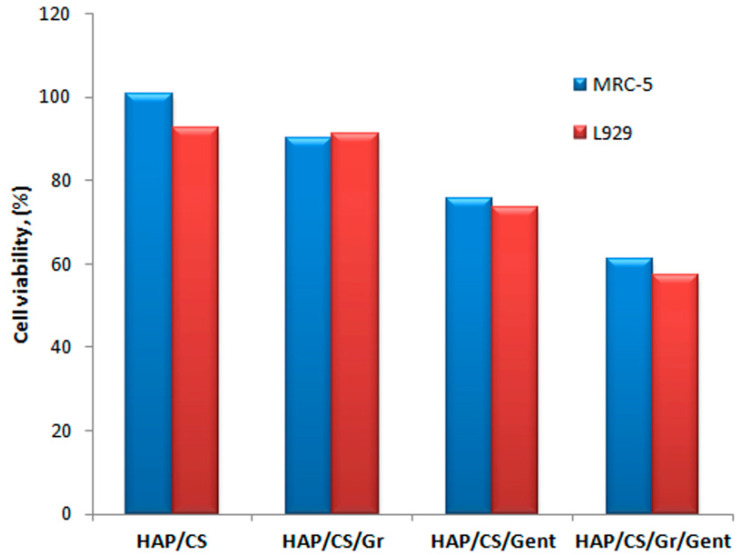
Cell viability of MRC-5 and L929 cell lines towards HAP/CS/, HAP/CS/Gr, HAP/CS/Gent and HAP/CS/Gr/Gent coatings. Adapted with permission from [72]. Copyright (2018) American Chemical Society. Adapted from [71]. Copyright (2020), with permission from Wiley.

MTT cytotoxicity test was utilized for the in vitro survival evaluation of the two targeted cell lines, MRC-5 (human fibroblasts) and L929 (mice fibroblasts) [71,72]. The results are shown in Figure 9, displaying cell viability in the presence of HAP/CS, HAP/CS/Gr, HAP/CS/Gent, and HAP/CS/Gr/Gent compared to the control (blank suspension containing only cells).

As per one of the cytotoxicity classification scales for biomaterials [116], there is no evidence to indicate cytotoxicity of HAP/CS (viability approx. 100%) and HAP/CS/Gr for both tested cell lines (viability > 90%), whereas HAP/CS/Gent and HAP/CS/Gr/Gent exhibited slight cytotoxicity (viability in the interval of 60−90%). The viability of L929 cells slightly decreased after incubation with both coatings, in comparison to MRC-5 (human fibroblasts) cells, which could be explained by the enhanced sensitivity of the mice cell line.

## 5. Conclusions

Bone implant materials are increasingly attracting attention in the biomaterials field. Among them, hydroxyapatite (HAP) stands out as a prospective biomaterial due to its excellent osseointegration ability, due to its similarity to natural bone. However, due to the lack of adhesive and antibacterial properties, it is usually combined with polymers and antibacterial agents. Natural polymers like lignin (Lig) and chitosan (CS) were proven as effective components of HAP-based composites, improving the adhesion and serving as a drug carrier. Inclusion of antibacterial agents into composite biomaterials has gained a lot of attention, as it is thus possible to achieve the desirable antibacterial activity. 

Composite antibacterial Ag/HAP, Ag/HAP/Lig, HAP/CS/Gent and HAP/CS/Gr/Gent coatings were obtained on titanium plates using electrophoretic deposition (EPD). The X-ray diffraction (XRD), field emission scanning electron microscopy (FE-SEM), Fourier transform infrared spectroscopy (FTIR), X-ray photoelectron analysis (XPS) and electrochemical impedance spectroscopy (EIS), performed after immersion in SBF solution at 37 °C, proved coatings bioactivity by formation and growth of “AB-type” carbonate-substituted HAP, known for its excellent bioactivity and similarity to the natural bone. Tests in suspension (quantitative monitoring changes in the viable number of bacterial cells) indicated strong antibacterial activity against *Escherichia coli* and *Staphylococcus aureus*. Biocompatibility was confirmed using in vitro MTT testing since a non-cytotoxic effect was shown towards healthy human peripheral blood mononuclear cells PBMC, human fibroblast cell line MRC-5 and mice fibroblast cell line L929, suggesting high potential for bone tissue engineering and medical applications.

## Figures and Tables

**Table 1:** Time dependence of coating pore resistance, *R*_p_, for Ag/HAP and Ag/HAP/Lig coatings during exposure to SBF at 37 °C.

Coating	*t*/h	*R*_p_/kΩ cm^2^
Ag/HAP	24	6.6
	120	11.7
	240	12.6
Ag/HAP/Lig	24	5.6
	120	5.9
	240	6.3

**Table 2:** Concentration of silver ion released from the Ag/HAP/Lig coating during exposure to SBF at 37 °C.

*t*/h	*c* (Ag^+^ Ion Released)/ppm
1	0.455
4	0.550
24	0648
48	0.802
72	0.881
120	1.060
144	1.294
168	1.371
192	1.486
216	1.557
240	1.704

**Table 3:** *Staphylococcus aureus* colony forming units (CFUs) in control and in suspensions incubated with Ag/HAP and Ag/HAP/Lig coatings and the survival of PBMC cells in the coating presence. (Adapted from [76]. Copyright (2013), with permission from Elsevier).

*S. aureus* Colonies Incubated with Coating Material				
Sample		Control	Ag/HAP	Ag/HAP/Lig
*S. aureus* [CFU mL^−^^1^]	Incubation period, h			
	0	1.0 × 10^5^	1.2 × 10^4^	2.5 × 10^4^
	1	3.0 × 10^4^	1.62 × 10^3^	2.0 × 10^3^
	24	9.9 × 10^4^	0	0
Survival of Peripheral Blood Mononuclear Cells (PBMC)				
Cell survival (S), %		100%	94.6 ± 4.2	89.4 ± 3.5
Classification		n/a	non-cytotoxic	non-cytotoxic
PHA-Stimulated Peripheral Blood Mononuclear Cells (PBMC)				
Cell survival (S), %		100%	92.1 ± 5.0	83.8 ± 6.3
Classification		n/a	non-cytotoxic	non-cytotoxic

**Table 4:** Mass of released Gent from HAP/CS/Gent and HAP/CS/Gr/Gent coatings during 21 days in deionized water at 37 °C.

Release Time (Days)	HAP/CS/Gent	HAP/CS/Gr/Gent
	Mass of Released Gent (μg)	
1	2.43	7.65
7	9.40	18.13
21	11.80	20.63

## Data Availability

Data sharing is not applicable to this article.
